# Research priorities in vulvodynia: A modified Delphi study

**DOI:** 10.1177/17455057251378957

**Published:** 2025-10-16

**Authors:** Athina Zoi Lountzi, Purva Abhyankar, Hannah Durand

**Affiliations:** 1 Division of Psychology, Faculty of Natural Sciences, University of Stirling, UK; 2 Usher Institute, College of Medicine and Veterinary Medicine, University of Edinburgh, UK

**Keywords:** vulvodynia, research prioritisation, expert engagement

## Abstract

**Background:**

Vulvodynia is a chronic, unexplained pain in and around the vulva, likely involving an interplay of biological and psychosocial factors. Women with vulvodynia often experience delayed diagnoses, ineffective treatments, and significant quality of life impacts, compounded by social stigma and negative healthcare experiences. Despite its prevalence, our understanding of vulvodynia and its impacts remains limited.

**Objectives:**

To establish research priorities that address critical knowledge deficits and improve outcomes for individuals affected by vulvodynia.

**Design:**

A mixed-methods participatory study using a modified electronic Delphi (e-Delphi) approach combined with focus groups.

**Methods:**

A three-phase modified e-Delphi process was combined with focus groups to gather insights from patients, clinicians, and researchers with expertise in vulvodynia. In Phase 1, participants generated research topics through surveys and focus group discussions. In Phase 2, these topics were rated and ranked by participants to generate a preliminary “top 10” list of priorities. In Phase 3, participants re-rated and re-ranked the preliminary list to achieve consensus on the final research priorities.

**Results:**

The top three priorities identified were: (1) Creating a person-centred care pathway and increasing awareness, education, and training of clinicians on vulvodynia, (2) Development of multidisciplinary pain teams, and (3) Creating accessible information for patients on treatment options and self-care advice.

**Conclusion:**

This study highlights the importance of integrating the perspectives of those with lived experience, healthcare professionals, and researchers to identify research priorities with the greatest potential for impact. Findings provide a roadmap for future vulvodynia research, support efficient resource allocation, and inform policy development. Furthermore, these results provide a foundation for grassroots initiatives to improve awareness, education, and care for individuals affected by vulvodynia.

## Plain language summary

What should researchers study next? Identifying the most important research questions about vulvodynia

Vulvodynia is a painful and often misunderstood condition that affects the vulva. People with vulvodynia often struggle to get a diagnosis, face limited and inconsistent treatment options, and experience stigma and dismissal from healthcare providers. Research on vulvodynia has increased, but it is unclear whether the most important issues for patients and clinicians are being addressed. To identify what research is most needed, we conducted a Delphi study, an expert-driven process that gathers opinions across multiple rounds to reach consensus on key priorities. We invited patients, healthcare providers, and researchers to participate in a three-stage online survey. Participants first generated a list of research topics they believed were most important. They then rated and ranked these topics, leading to a final list of the top 10 research priorities for vulvodynia. The top three research priorities identified were:

1. Creating a person-centered care pathway and improving clinician awareness, education, and training.

2. Developing multidisciplinary pain teams that include gynecologists, physiotherapists, and mental health professionals.

3. Providing accessible patient resources on treatment options and self-care strategies.

Other important research topics included understanding the causes of vulvodynia, mental health support, diagnostic tools, and ways to raise public awareness. **Key Takeaway:** Patients and clinicians agree that better education for healthcare providers, improved care pathways, and multidisciplinary support are top priorities for vulvodynia research. These findings provide a roadmap for future research, helping guide funding, policy decisions, and healthcare improvements.

## Background

Vulvodynia is defined as an idiopathic chronic pain condition affecting the vulva. According to the International Society for the Study of Vulvovaginal Disease, it involves persistent vulvar pain lasting at least 3 months, with no clear identifiable cause and possibly influenced by other contributing factors.^1^ There is a lack of epidemiological research to determine the worldwide impact of vulvodynia; however, it is estimated to affect approximately 10%–28% of women of reproductive age.^2^ These rates are thought to be an underestimation as vulvodynia is commonly misdiagnosed as other conditions such as vaginismus, persistent yeast infections, and urinary tract infections.^3^

The aetiology of vulvodynia is unknown, though it is thought to involve a multifactorial interplay of physiological and psychosocial factors.^4,5^ Women with vulvodynia often experience symptoms for months or even years before receiving a diagnosis.^6^ Managing vulvodynia presents further challenges, as there are no standardised guidelines for its management, and treatment approaches vary widely. Options include topical treatments (e.g. lidocaine, gabapentin), oral medications (e.g. tricyclic antidepressants), self-care techniques, physiotherapy (e.g. pelvic floor therapy, soft tissue work), psychotherapy, and surgical interventions.^7^

Vulvodynia is a chronic pain condition shaped by unique social pressures and negative experiences in seeking medical care. A lack of awareness and education about the condition contributes to stigma, confusion, and embarrassment among those affected.^8^ Research highlights a significant gap in awareness among healthcare professionals,^9,10^ with many women reporting feeling unheard and dismissed, and having their symptoms and pain experiences minimised and misunderstood.^11,12^ Vulvodynia can also negatively affect sexual and relationship satisfaction, particularly when compounded by heteronormative sexual scripts and societal expectations.^13^ Therefore, vulvodynia represents a multidimensional health issue with profound physiological, psychological, and social consequences. Advancing research on vulvodynia requires a comprehensive understanding of its complexity and the diverse factors influencing its onset, duration, and management.

Women living with vulvodynia possess unique and valuable expertise, developed through first-hand experiences of persistent symptoms, delayed diagnoses, and the pursuit of effective treatment in the face of systemic barriers.^14^ This experiential knowledge aligns with feminist epistemological frameworks (e.g. standpoint theory), which recognise individuals as experts of their own bodies and value embodied knowledge as a legitimate and necessary foundation for research and practice.^15–18^ Despite this, they are rarely, if ever, included in the development of research projects or the establishment of research priorities. Their involvement is typically limited to participation as survey or interview respondents, restricting their ability to shape research agendas meaningfully. Similarly, primary care practitioners and clinicians, often the first point of contact for women with vulvodynia, have limited opportunities to share their insights on the condition or the challenges of delivering care.^14,19^ It remains unclear whether existing research adequately addresses the full spectrum of issues faced by women affected by vulvodynia or those providing care. A recent scoping review of clinical and psychosocial research on vulvodynia further highlighted these gaps, identifying inconsistent methodologies, limited patient involvement, and a lack of research on care access and lived experience.^20^ Hence, it is essential to engage not only women with lived experience but also healthcare practitioners and other key experts with expertise in vulvodynia to gain a more comprehensive understanding of the condition and its associated challenges.^21^ This study, therefore, aims to identify and achieve consensus on research priorities for vulvodynia among key experts. By highlighting research targets with the greatest potential for impact, the study seeks to promote a holistic understanding of the research needs. These findings could enhance care, advance scientific understanding, and ultimately provide better support for individuals affected by vulvodynia.

## Methods

The study employed a mixed-methods participatory research approach, combining focus groups with an electronic Delphi (e-Delphi) approach. In line with previous research prioritisation exercises,^22,23^ the e-Delphi approach involved online administration of iterative questionnaires. Through this method, feedback is systematically gathered and consensus is achieved over multiple rounds, with participants receiving feedback on the group’s responses from previous rounds. This iterative process allows for thoughtful reflection and refinement of input. This approach has been demonstrated to be well-suited for engaging large and geographically dispersed participant groups, enhancing accessibility and inclusivity.^24–26^ Ethical approval was obtained from the University of Stirling General University Ethics Panel (GUEP 2024 17830 13489). The Consolidated Criteria for Reporting Qualitative Research guidelines^27^ were used to guide the reporting of this study (see Supplemental File A).

### Sample

In line with participatory research principles, we use the term “experts” to refer to individuals with lived, clinical, or academic expertise related to vulvodynia. This inclusive definition reflects the study’s epistemological stance that values different forms of knowledge (i.e. experiential, clinical, and research-based) as equally important in shaping research priorities. Participants were purposively selected according to the following criteria, which defined the key experts of the study:

1. Individuals with a formal diagnosis of vulvodynia from a healthcare professional
2. Healthcare professionals with experience in providing care to patients with vulvodynia
3. Researchers with expertise in vulvodynia
4. Individuals working with or on behalf of people with vulvodynia, for example, through a charity or support organisation.

Potential participants were identified through social media platforms, online support groups, professional networks (e.g. British Society for the Study of Vulval Disease), and support organisations (e.g. Vulval Pain Society). Snowball sampling was employed, whereby participants were encouraged to share the study with peers or colleagues who may also be eligible to take part. Participants from the United States, Australia, and Europe were eligible to participate if they could complete the study in English, with most participants based in the United Kingdom, United States, and Australia. Eligible participants accessed the study via a survey link, where they could provide their email address to participate in subsequent research stages and focus group discussions.

### Data collection

Data were collected using the SurveyMonkey platform, allowing the study to be conducted entirely online and include participants from multiple countries. Three rounds of surveys were administered in conjunction with focus groups. Focus groups were conducted online, allowing participants to maintain their anonymity and increasing the accessibility of the research. Responses from the initial questionnaire and focus groups guided the development of the Phases 2 and 3 questionnaires. Electronic reminders were sent at the start and toward the end of each phase to encourage participation. To further incentivise engagement, participants responding in Phase 3 were given the option to enter a prize draw for 1 of 10 £25 Amazon vouchers. Data collection took place between April and September 2024. All participants provided informed consent via a secure online form prior to taking part in the study. Considering the potential limitations of online recruitment strategies, efforts were made to ensure data quality by allowing only one entry per IP address, manually reviewing free-text responses, and performing data cleaning procedures to remove incomplete or nonsensical responses. No free-text responses were excluded or omitted during data processing.

#### Phase 1

Phase 1 concerned the experts’ topic generation. Experts were asked to suggest research questions and/or topics they considered important for vulvodynia via free-text response. They were prompted with the question: “In your opinion, what are the most important questions or problems related to vulvodynia that research could address? What are your reasons for selecting this/these question(s) or problem(s)?” The full Phase 1 survey is available as an appendix (see Supplemental File B). The Phase 1 survey was open for 4 weeks.

##### Focus groups

To complement the survey, experts were invited to participate in online focus group discussions to gather more in-depth information on the proposed research topics. This dual approach enriched the topic generation phase by enabling experts to contribute their insights through multiple channels. Focus groups were conducted online by A.Z.L., a female health psychology researcher with training and experience in qualitative methods and vulvodynia research. Participants maintained anonymity by using pseudonyms. Discussions followed a semi-structured format, encouraging open and free-flowing dialogue. The following questions were used as a topic guide to facilitate the conversation:

1. What are the most significant challenges you or others you know have faced in managing vulvodynia, personally or in practice?
2. Based on your experience or expertise, what do you believe are the key gaps in our understanding of vulvodynia?
3. Are there particular aspects of vulvodynia (e.g. aetiology, diagnosis, treatment, impact on quality of life) that you believe require more attention?

Focus groups were audio-recorded and transcribed verbatim, with field notes taken during sessions. Each focus group lasted approximately 20 min. Data saturation was considered achieved when no new themes were identified from the third focus group, with participant responses reflecting repetition of topics raised in previous discussions.

#### Phase 2

Phase 2 involved the administration of an e-Delphi online survey developed using data from the Phase 1 survey and focus groups. Participants who took part in Phase 1 were invited to complete the Phase 2 survey. Recruitment was expanded by advertising Phase 2 on social media platforms and professional networks to attract additional participants.

In this phase, participants were presented with a comprehensive list of research topics generated in Phase 1. They rated the importance of each topic on a 10-point scale (1 = lowest importance; 10 = highest importance). After completing their ratings, participants were asked to select and rank their “top 10” most important topics. To accommodate the perspectives of new participants, a free-text box was included to allow for additional topic suggestions. Phase 2 was conducted over 4 weeks.

#### Phase 3

Phase 3 marked the final step in the e-Delphi process, focusing on refining and achieving consensus on the research priorities identified in Phase 2. Participants who took part in Phase 2 were provided with summary information on how others had rated and ranked the research topics. Using this feedback, participants were asked to re-rate the research topics, taking into account the aggregated results. They were also shown the mean ranking scores for the top 10 research topics identified in Phase 2 and asked to re-rank their own top 10 priority items. This phase was open for 6 weeks.

### Analysis plan

IBM SPSS Statistics 29 was used to analyse the data. Descriptive statistics were calculated for demographic data, including details about the respondents’ expertise during Phase 1 and Phase 2. In Phase 1, all suggested free-text research priorities were collated and categorised to create a comprehensive list of potential topics. Duplicate responses were merged to minimise overlap in subsequent phases. Focus group discussions were transcribed and analysed using an inductive thematic analysis approach to identify additional research topics not reflected in the survey data.^28^ In Phase 2, descriptive statistics were calculated for demographic data, topic ratings, and priority rankings. The mean rating of each topic was computed to evaluate participants’ perceptions of importance, and these values were displayed during Phase 3. To determine the top 10 priorities, the mean rankings of topics were calculated using the Wilcoxon Signed-Rank Test and presented to participants in Phase 3. Following Phase 3, the mean re-ratings and re-rankings of priorities were calculated to finalise the consensus on research priorities.

## Results

### Participant characteristics

One hundred and six participants responded to the Phase 1 survey, with 21 of these participants also taking part in the focus group discussions. One hundred and four participants took part in Phase 2, and 73 participants took part in Phase 3. Participant demographics are displayed in Table 1.

**Table 1.** Participant characteristics.

	Phase 1	Phase 2	Phase 3
Variable	*n* (%)	*n* (%)	*n* (%)
Respondents	106 (100)	104 (100)	73 (100)
Patients	92 (86.8)	66 (63.5)	57 (78.1)
Healthcare professionals	9 (8.5)	6 (5.8)	6 (8.2)
Researchers	4 (3.8)	4 (3.9)	2 (2.7)
Other	4 (3.8)	1 (1.0)	1 (1.4)
Mean age (SD)	43.1 (15.5)	43.6 (15.3)	44.3 (15.3)
Country of residence			
UK	22 (20.8)	31 (29.8)	28 (38.4)
USA	20 (18.9)	22 (21.2)	20 (27.4)
Australia	13 (12.3)	8 (7.7)	8 (11.0)
New Zealand	7 (6.6)	6 (5.8)	4 (5.5)
Canada	4 (3.8)	3 (2.9)	3 (4.1)
Other European country	4 (3.8)	2 (1.9)	1 (1.4)
Not reported	36 (34.0)	32 (30.8)	9 (12.3)

SD: standard deviation.

*Note.* Some participants identified with more than one category; therefore, columns may add up to more than 100%.

### Research prioritisation

#### Phase 1

In *Phase 1*, participants’ survey responses were collated and categorised to identify research topics. In total, 29 research topics were generated in *Phase 1* from both the survey and focus group data. Five topic themes were identified from the focus group data: (1) Multidisciplinary pain teams; (2) Recognised pathway of care; (3) Healthcare professional awareness; (4) Vulvodynia aetiology; (5) Comorbidities. Although no unique topics were identified from the focus group data, they provided deeper contextual insights into the themes identified through the survey. This additional qualitative data helped refine and expand the understanding of each research topic. Illustrative quotes from the focus groups are presented in Table 2. These quotes offer further insight into the emotional and experiential dimensions of vulvodynia, which may inform future patient-centred research. The full list of *Phase 1* topics can be found in Table 3.

**Table 2.** Illustrative FG participant quotes by theme.

Theme	Illustrative quote
Multidisciplinary pain teams	“There need to be specialists on vulvodynia. [. . .] It would be really good if there was some sort of, like, medical specialists who were a pelvic chronic pelvic floor dysfunction, pain person to see.” (Patient, FG2)
	“Dealing with the pain is stressful. [. . .] The mental health aspect, it knocks you as a woman. [. . .] You get a diagnosis, medications, but no one is thinking about how it affects your broader life [. . .] very little support in mental health.” (Patient, FG1)
	“Specialists don’t seem to speak to one another. They keep referring to other professionals. Why can’t they be in the same room at the same time? It felt like I just kept being passed around.” (Patient, FG1)
Recognised pathway of care	“There needs to be specialist clinics about vulvodynia. [. . .] I don’t know where to find help for my pain. [. . .] I hated when practitioners told me everything looked great when they really didn’t know how to treat vulvodynia or where to refer me.” (Patient, FG2)
	“There needs to be a compiled list of doctors in each state (USA) who know about it. [. . .] There isn’t much integration between GPs and sexual health clinics.” (Clinician, FG3)
Healthcare professional awareness	“Doctors, especially gynaecologists, should be given a lot more training in women’s vulval pain conditions. [. . .] They hardly receive any training in this area at all, most doctors have never heard of this before. [. . .] It is exhausting fighting on your own.” (Patient, FG1)
	“I have tried a few different things as treatments. [. . .] There doesn’t seem to be a standard treatment, and I wonder why. I was on various contraceptive pills for years and these could have been making the issue worse for me, but nobody ever flagged it in primary care.” (Patient, FG3)
	“Doctors do not seem to understand the level of pain [. . .] [They] put pressure on patients to have vaginal sex, [. . .] are judgmental of our sexual lives, of whether we want kids, et cetera.” (Patient, FG1).
Vulvodynia aetiology	“I’ve had many doctors who tell me vulvodynia is a nerve disorder even though by definition the cause is unknown, or some doctors who stop looking for solutions once a diagnosis of vulvodynia is given. [. . .] If they knew what caused it then we could hopefully prevent it in the first place.” (Patient, FG2)
	“More research needed on the mechanism or cause on a neurological level. The physical cause of vestibulodynia has been show in histological research, the actual mechanisms that cause the pain seem to be poorly understood and treatment is often on trial/error basis [. . .] identifying different types, mechanism and causes of vulvodynia.” (Clinician and researcher, FG3)
Comorbidities	“I think the link between inflammatory conditions like Crohn’s disease and ankylosing spondylitis needs to be explored. I had one provider tell me there was a link, but no one seems to know.” (Patient, FG4)
	“I have several other issues, some of which I strongly believe are related and others that may have an influence. I have had full-body nerve pain and muscle pain for also about ten years, [. . .] suspected [attention-deficit/hyperactivity disorder], [. . .] and all may potentially be related to fluctuations in dopamine nerves and pain perceptions. Furthermore, I have [polycystic ovary syndrome], which may not be directly related but definitely has an influence on the pain level.” (Patient, FG1)

FG: focus group.

**Table 3.** Summary of research topics identified in phase 1 with participants’ ratings of importance from phases 2 and 3.

Research topic	Phase 2	Phase 3
	Mean (SD)	Mean (SD)
Creating person-centred care pathways and increasing awareness, education, and training of clinicians on vulvodynia	9.38 (1.1)	9.49 (0.7)
Underlying causes and trigger factors	8.75 (1.64)	9.03 (1.15)
Recognition of vulvodynia as a potentially disabling condition	8.64 (1.82)	8.45 (1.54)
Effective dissemination of information to patients on treatment options and self-care advice	8.59 (1.64)	9.01 (1)
New treatments for vulvodynia	8.57 (1.77)	8.87 (1.2)
Development of multidisciplinary pain teams for vulvodynia	8.54 (1.93)	9.13 (1.02)
Development of accessible information for patients on treatment options and self-care advice	8.47 (1.79)	8.57 (1.5)
Identifying standard clinical and patient-reported outcome measures	8.01 (1.98)	8.36 (1.16)
Patient stories and experiences on successful management of vulvodynia	7.78 (2.17)	7.66 (1.76)
Mental health support	7.77 (2.24)	8.21 (1.66)
Effective ways of raising public knowledge and awareness about vulvodynia	7.76 (2.12)	7.85 (1.41)
Establishment and validation of diagnostic subcategories based on aetiology	7.73 (2.25)	8.21 (1.26)
Association between the vaginal microbiome and vulvodynia	7.68 (2.15)	7.69 (1.52)
Development of online resources on help-seeking for vulvodynia	7.53 (2.14)	7.82 (1.53)
Vulvodynia prevention	7.53 (2.65)	7.81 (1.91)
Role of hormones in vulvodynia	7.51 (2.32)	7.96 (1.45)
Role of menopause in vulvodynia	7.47 (2.25)	7.90 (1.68)
Role of comorbidities in vulvodynia	7.45 (2.1)	8.01 (1.38)
Role of the musculoskeletal system in vulvodynia	7.40 (2.13)	7.75 (1.3)
Effective peer support (online/in-person, group/individual)	7.37 (2.34)	7.78 (1.6)
Vulvodynia relapse	7.32 (2.1)	7.68 (1.62)
Role of inflammation in vulvodynia	7.01 (2.25)	7.59 (1.45)
Role of psychological and/or physical trauma in vulvodynia	6.93 (2.36)	7.15 (1.83)
Role of pregnancy and childbirth in vulvodynia	6.93 (2.33)	7.55 (1.61)
Sociodemographic factors in vulvodynia	6.91 (2.61)	6.76 (1.99)
Role of antibiotics in vulvodynia	6.90 (2.68)	7.09 (1.74)
Role of diet in vulvodynia	6.78 (2.52)	6.81 (2.01)
Sex therapy as a treatment for vulvodynia	6.72 (2.48)	6.88 (2.19)
Support for partners	6.07 (2.53)	6.91 (2.38)

SD: standard deviation.

*Note.* Participants were presented with the topics and were asked to rate the importance of each topic on a rating scale of 1 (least important) to 10 (most important). The mean importance of each topic as rated by the participants is displayed in the table.

#### Phase 2

In *Phase 2*, the mean ratings of importance were calculated for the research topics identified in Phase 1. The mean importance ratings of each topic are shown in Table 3. A Wilcoxon signed-rank test was used to analyse the rank data and calculate the mean rankings of the preliminary “top 10” research priorities. The top three ranked research topics in Phase 2 were: (1) “Development of a multidisciplinary pain team (involving GPs, gynaecologists, physiotherapists, nurses, sexual health specialists),” (2) “Creating a person-centred care pathway and increasing awareness, education, and training of clinicians on vulvodynia,” and (3) “Development of accessible information for patients on treatment options and self-care advice.”

#### Phase 3

In *Phase 3*, participants rated the following research topics as most important: “Creating a person-centred care pathway and increasing awareness, education, and training of clinicians on vulvodynia”; “Development of a multidisciplinary pain team (involving GPs, gynaecologists, physiotherapists, nurses, sexual health specialists) for management of vulvodynia”; “Vulvodynia underlying causes and trigger factors.” Table 3 presents the mean re-ratings of research topics. After reviewing Phase 2 ratings, participants increased their importance ratings for most priorities in Phase 3, except for three topics, which were rated slightly lower (i.e. “Recognition of vulvodynia as a potentially disabling condition,” “Patient stories and experiences on successful management of vulvodynia,” and “Sociodemographic factors in vulvodynia”). A Wilcoxon signed-rank test was used to calculate the mean rankings of the “top 10” research priorities. The final consensus on research priorities and their mean ranking scores are presented in Table 4. While the top three priorities remained unchanged, “Creating a person-centred care pathway and increasing clinician awareness and training” emerged as the highest-ranked priority in Phase 3.

**Table 4.** Final consensus on the top 10 research priorities for vulvodynia.

Research topic	Mean ranking score (SD)
Creating person-centred care pathways and increasing awareness, education, and training of clinicians on vulvodynia	1.82 (1.05)
Development of multidisciplinary pain teams for vulvodynia	2.12 (1.62)
Development of accessible information for patients on treatment options and self-care advice	3.91 (1.73)
Effective dissemination of information to patients on treatment options and self-care advice	5.09 (1.55)
Development of online resources on help-seeking for vulvodynia	5.17 (1.69)
Identifying standard clinical and patient-reported outcome measures	6.04 (2.37)
Mental health support	7.25 (1.83)
Vulvodynia prevention	7.28 (2.06)
Establishment and validation of diagnostic subcategories based on aetiology	7.44 (2.58)
Effective peer support (online/in-person, group/individual)	8.88 (1.36)

SD: standard deviation.

*Note.* Participants selected their top 10 topics. The mean ranking of each topic, as determined by the participants, is shown above, ordered from highest to lowest ranked.

## Discussion

This study identified and prioritised top research topics for vulvodynia through a participatory e-Delphi process involving patients, clinicians, and researchers. The findings highlight the most pressing needs in vulvodynia research, including the development of person-centred care pathways, multidisciplinary pain teams, and greater understanding of vulvodynia’s underlying causes and triggers. Conversely, topics such as partner support, sex therapy, and dietary influences were rated as lower priorities, reflecting participants’ emphasis on systemic healthcare challenges rather than individual lifestyle factors. By integrating diverse perspectives and achieving consensus, this study provides a roadmap for future research that addresses the multidimensional challenges of vulvodynia, with the potential to improve care, awareness, and outcomes for those affected.

### Developing a person-centred pathway of care

The highest-rated research priority was the development of a person-centred care pathway for vulvodynia. This includes establishing clear pathways for seeking and delivering care while enhancing clinician awareness, education, and training. Previous research on help-seeking experiences has consistently highlighted the negative and often dismissive responses women encounter when seeking care for vulvodynia.^11,12^ Women’s pain, particularly gynaecological pain, is frequently underestimated or downplayed, leading to significant delays in diagnosis and treatment. Many women report having to advocate for themselves to receive appropriate care, reflecting pervasive gender biases in healthcare. These biases often result in women’s symptoms being dismissed or attributed solely to psychological factors. A lack of awareness and education among healthcare professionals further compounds these challenges, leaving women without the support or understanding they need.^11,25^ Addressing this requires the development of best practice guidance, the creation of targeted educational resources, and the integration of these efforts into healthcare policy. Ensuring that healthcare professionals are equipped with accurate, evidence-based knowledge can improve help-seeking experiences, reduce diagnostic delays, and foster more compassionate, effective care.

### Establishing multidisciplinary pain teams

The second-highest priority identified was improving access to multidisciplinary pain teams. Given the complex, multifactorial nature of vulvodynia, a collaborative treatment approach involving gynaecologists, physiotherapists, pain specialists, and mental health professionals has been widely advocated.^4^ Multidisciplinary teams can deliver tailored treatment pathways that address the diverse symptoms and needs of patients. Integrating multidisciplinary teams within a structured, person-centred care framework would ensure clear referral routes, reduce fragmentation, and encourage interdisciplinary collaboration. This priority highlights the need for further research into how best to implement and evaluate multidisciplinary models of care for vulvodynia. It also aligns with best practice models for other chronic pain conditions, suggesting that coordinated, multimodal interventions may yield better patient outcomes.

### Developing reliable patient resources and raising awareness

Another top priority was the development of accessible and reliable resources for patients. These resources should provide clear guidance on where to seek help, self-care advice, and educational materials in lay language, based on frequently updated peer-reviewed guidelines. Links to support organisations should also be encouraged, empowering women with tools and information to feel supported before consulting healthcare providers. Previous research highlights inconsistencies and a lack of reliable online information about vulvodynia, contributing to confusion, misinformation, and uncertainty about where to seek help or whom to contact.^29^ There is a need for studies on effective dissemination strategies to ensure accurate, accessible, and widely available information. Developing clear, evidence-based, patient-friendly resources could promote healthcare-seeking behaviours and reduce barriers to care.

This priority aligns with public awareness efforts, which aim to reduce stigma and increase recognition of vulvodynia as a potentially disabling condition. Research into the impact of vulvodynia on daily activities and work productivity is essential to better understand the condition’s broader consequences. Such studies could provide critical evidence to advocate for workplace accommodations and policy reforms, reducing stigma and discrimination. Enhanced awareness and institutional support would not only improve the professional and social well-being of affected women but also contribute to a more inclusive and supportive environment for managing chronic health conditions.

### The role of peer support and mental health services

Peer support was also identified as a critical priority. Participants emphasised the value of facilitating connections to existing support groups and creating local communities—both online and in-person—for women to share experiences. While peer support has proven beneficial in managing chronic pain conditions like cancer and diabetes, its role in lesser-known conditions such as vulvodynia remains underexplored.^30^ Peer support has been shown to improve emotional well-being and coping strategies in chronic pain management,^30^ and further research is needed to evaluate its potential benefits for vulvodynia patients. This includes exploring its effects on reducing isolation, improving access to resources, and identifying effective methods for implementing such networks.

Additionally, mental health support was identified as one of the top priorities. Participants emphasised the importance of connecting patients with mental health professionals specialising in chronic pain and providing links to mental health organisations to address the psychological burden associated with vulvodynia. One promising avenue for future research could involve integrating studies on peer and mental health support. This approach could provide a more comprehensive understanding of how patients can be supported both through clinical healthcare interventions and social networks, ensuring holistic care that addresses both emotional and physical well-being.

### Exploring new diagnostic and treatment pathways

Research into novel treatment pathways, such as genetic and connective tissue studies, was a highly rated priority. These underexplored areas hold significant potential for uncovering the underlying mechanisms of vulvodynia, ultimately informing new therapeutic strategies. Recent studies have begun to explore the role of genetic factors in vulvodynia, identifying specific genetic predispositions and familial patterns that may contribute to the condition’s aetiology.^31–33^ The heterogeneity of vulvodynia presentations suggests that personalised treatment strategies may be more effective than one-size-fits-all approaches. Investigating the biological aspects of vulvodynia—such as genetic, neurological, and inflammatory factors—is essential for understanding its mechanisms and creating targeted, individualised therapies, particularly for patients who do not respond to current treatment options.

Participants also identified the need to establish and validate diagnostic subcategories for vulvodynia. Such subcategories could facilitate more accurate diagnoses and personalised treatment options. For example, proposed diagnostic categories such as hormonally associated vestibulodynia, inflammation, congenital or acquired neuroproliferation, and hypertonic pelvic floor muscle dysfunction have shown promise in clinical settings for guiding diagnosis and treatment.^34,35^ However, further research is needed to validate and refine these classifications, ensuring they effectively guide clinicians in diagnosing and treating vulvodynia.^36^

### Standardising outcome measures

A major barrier to progress in vulvodynia research is the lack of standardised clinical and patient-reported outcome measures. Currently, the lack of consensus on clinical and patient-reported outcome measures limits the comparability of research findings and hinders translation of evidence into practice. Participants emphasised the need to define and adopt standardised outcome measures to enhance the rigour and practical impact of future studies. Importantly, achieving consensus on these measures should include the perspectives of patients to ensure they capture meaningful aspects of their experiences.

The multifactorial nature of vulvodynia suggests that multiple outcome measures are likely required to reflect its complex impact on patients. Standardised measures would enable consistent evaluation of treatment effectiveness, efficacy, and feasibility across various modalities. This consistency is essential for identifying the most effective interventions and ensuring that evidence can be reliably applied in clinical settings. Additionally, standardisation would improve clinical decision-making and support the delivery of higher-quality care for vulvodynia patients.^36,37^

### Implications for research and practice

The findings of this study highlight critical research priorities for advancing the understanding and management of vulvodynia. However, these priorities do not necessarily reflect a lack of existing research but may indicate a gap in the effective implementation of research evidence into clinical practice. This underscores the need for translational research and knowledge dissemination strategies to ensure that evidence informs clinical practice and policy effectively. Implementation science frameworks, such as the Knowledge-to-Action model, could be applied to bridge this gap by identifying key barriers to integrating evidence-based guidelines into routine clinical care. Addressing this implementation gap is critical to advancing care for individuals with vulvodynia and ensuring that research findings have a tangible impact on patient outcomes. Future research should focus not only on addressing the identified priorities but also on developing strategies to translate evidence into actionable care pathways. By fostering collaboration among researchers, clinicians, and policymakers, these efforts can enhance clinical decision-making, improve patient outcomes, and support the delivery of holistic, patient-centred care for individuals with vulvodynia.

### Strengths and limitations

This modified e-Delphi study demonstrates its value as a method for identifying emerging research priorities and areas requiring increased focus. The dual-method approach ensured a systematic, iterative, and in-depth exploration of research priorities by combining breadth from surveys with depth from focus groups, thereby enhancing the richness and quality of the data. The iterative e-Delphi process, which included sharing aggregated feedback with participants in each round, fostered transparency and ensured that consensus was built collaboratively. The online nature of this methodology offered several advantages, including flexibility, anonymity, and accessibility for participants across diverse geographic locations.^21,22^ Inclusion of participants from multiple countries provided a global perspective on the challenges and priorities related to vulvodynia, increasing the generalisability and relevance of findings across different healthcare systems. Nevertheless, several limitations warrant consideration.

First, high attrition rates, a common challenge in Delphi studies, were evident in this research, with a 29% decrease in participation during the final round. This may reflect a retention bias, where participants who remained engaged were more likely to be those with stronger pre-existing opinions about vulvodynia research. As a result, lower-ranked topics (e.g. diet, partner support) may have received less attention not because they lack importance, but because they resonated less with the final sample. The dual task of rating all items and identifying the top 10 priorities, coupled with the large number of items to assess (*n* = 29), likely contributed to participant burden. Although the e-Delphi questionnaires were adapted from prior Delphi studies, they were not formally validated; however, they were pilot tested with five patient partners (i.e. approximately 5% of the Phase 1 sample) to improve clarity and accessibility. Despite these challenges, including all proposed items from Phase 1 was essential in providing a comprehensive perspective on experts’ needs during the priority-setting process. Future research should explore strategies to enhance retention across Delphi rounds, such as staggered surveys with embedded reminders and tailored incentives.

Second, we faced challenges in recruiting a diverse range of experts, with an underrepresentation of expert groups beyond patients in the final sample. It remains unclear whether the ratings provided by other expert groups differed from those of patients, as some participants identified with multiple categories, and others did not disclose demographic information. Although patients made up the majority of participants, their feedback revealed a critical concern: the limited awareness of vulvodynia among other key groups, particularly healthcare professionals. This lack of awareness may have restricted broader engagement. Equally, it is possible that non-specialist clinicians (e.g., GPs, nurses) who encounter vulvodynia patients may not have recognised themselves as sufficiently “expert” in the topic. This highlights the importance of addressing the top priorities identified in this study to raise awareness and foster greater involvement in vulvodynia research across diverse expert groups.

Finally, the online format may have disproportionately reflected the perspectives of patients from specific socioeconomic backgrounds, potentially limiting the diversity of experiences captured. While topics such as racial and socioeconomic differences, partner support, and the role of diet were rated as lower priorities, this may reflect the perspectives of the study’s participants rather than their overall importance. The predominance of patient participants, primarily based in the United Kingdom and United States, may have influenced the prioritisation of topics, particularly those concerning access to care. Although no consistent regional or socioeconomic trends were evident in the responses, it is possible that individuals from diverse geographic and cultural backgrounds were underrepresented. Nevertheless, the study’s overarching findings, including the need to raise awareness of vulvodynia, enhance public understanding, and establish a structured pathway of care, reveal that even fundamental aspects of help-seeking and care provision remain unmet for individuals with this condition.

## Conclusion

This study has identified the top research priorities for vulvodynia based on insights from key experts, ensuring future research aligns with the needs of those with lived experience. Grounding research priorities in expert needs has the potential to refocus efforts, catalyse meaningful change, and strengthen support for individuals with vulvodynia, while advancing the broader field of women’s health research. By highlighting these priorities, this work aims to guide researchers and clinicians toward addressing the most urgent challenges, optimising resource allocation, and ultimately enhancing the quality of life and care for individuals affected by vulvodynia. Additionally, the prioritisation findings can serve as a resource for funding bodies and policymakers, helping to guide future calls for research grants that align with real-world needs. Policymakers should consider supporting initiatives that integrate patient-centred care pathways into national clinical guidelines, ensuring that vulvodynia management receives structured attention within health systems. Future research efforts should centre on these identified needs to influence policy and drive initiatives that foster a more impactful understanding of vulvodynia and effective support for those affected.

## Supplemental Material

sj-pdf-1-whe-10.1177_17455057251378957 – Supplemental material for Research priorities in vulvodynia: A modified Delphi study Supplemental material, sj-pdf-1-whe-10.1177_17455057251378957 for Research priorities in vulvodynia: A modified Delphi study by Athina Zoi Lountzi, Purva Abhyankar and Hannah Durand in Women’s Health

sj-pdf-2-whe-10.1177_17455057251378957 – Supplemental material for Research priorities in vulvodynia: A modified Delphi study Supplemental material, sj-pdf-2-whe-10.1177_17455057251378957 for Research priorities in vulvodynia: A modified Delphi study by Athina Zoi Lountzi, Purva Abhyankar and Hannah Durand in Women’s Health
