# Supplementary material for: Research priorities in vulvodynia: A modified Delphi study
Source: Womens Health (Lond). 2025 Oct 16;21:17455057251378957. doi: 10.1177/17455057251378957 (PMC12536094; doi:10.1177/17455057251378957)
Supplement: sj-pdf-1-whe-10.1177_17455057251378957 – Supplemental material for Research priorities in vulvodynia: A modified Delphi study [file sj-pdf-1-whe-10.1177_17455057251378957.pdf]

Consolidated criteria for reporting qualitative studies (COREQ): 32-item checklist

No. Item	Guide questions/description	Reported on Page #
**Domain 1: Research team and reflexivity**		
Personal Characteristics		
1. Interviewer/facilitator	Which author/s conducted the interview or focus group?	5
2. Credentials	What were the researcher's credentials? E.g. PhD, MD	5
3. Occupation	What was their occupation at the time of the study?	5
4. Gender	Was the researcher male or female?	5
5. Experience and training	What experience or training did the researcher have?	5
Relationship with participants		
6. Relationship established	Was a relationship established prior to study commencement?	n/a
7. Participant knowledge of the interviewer	What did the participants know about the researcher? e.g. personal goals, reasons for doing the research	5
8. Interviewer characteristics	What characteristics were reported about the interviewer/facilitator? e.g. Bias, assumptions, reasons and interests in the research topic	5

**Domain 2: study design**		
Theoretical framework		
9. Methodological orientation and Theory	What methodological orientation was stated to underpin the study? e.g. grounded theory, discourse analysis, ethnography, phenomenology, content analysis	6
Participant selection		
10. Sampling	How were participants selected? e.g. purposive, convenience, consecutive, snowball	4
11. Method of approach	How were participants approached? e.g. face-to-face, telephone, mail, email	4
12. Sample size	How many participants were in the study?	6
13. Non-participation	How many people refused to participate or dropped out? Reasons?	n/a
Setting		
14. Setting of data collection	Where was the data collected? e.g. home, clinic, workplace	5
15. Presence of non-participants	Was anyone else present besides the participants and researchers?	n/a
16. Description of sample	What are the important characteristics of the sample? e.g. demographic data, date	7
Data collection		
17. Interview guide	Were questions, prompts, guides provided by the authors? Was it pilot tested?	5
18. Repeat interviews	Were repeat interviews carried out? If yes, how many?	n/a
19. Audio/visual recording	Did the research use audio or visual recording to collect the data?	5
20. Field notes	Were field notes made during and/or after the interview or focus group?	5
21. Duration	What was the duration of the interviews or focus group?	5
22. Data saturation	Was data saturation discussed?	5
23. Transcripts returned	Were transcripts returned to participants for comment and/or correction?	n/a

**Domain 3: analysis and findings**		
Data analysis		
24. Number of data coders	How many data coders coded the data?	6
25. Description of the coding tree	Did authors provide a description of the coding tree?	8
26. Derivation of themes	Were themes identified in advance or derived from the data?	6
27. Software	What software, if applicable, was used to manage the data?	n/a
28. Participant checking	Did participants provide feedback on the findings?	n/a
Reporting		
29. Quotations presented	Were participant quotations presented to illustrate the themes/findings? Was each quotation identified? e.g. participant number	8
30. Data and findings consistent	Was there consistency between the data presented and the findings?	8
31. Clarity of major themes	Were major themes clearly presented in the findings?	8
32. Clarity of minor themes	Is there a description of diverse cases or discussion of minor themes?	n/a
