# Supplementary material for: Research priorities in vulvodynia: A modified Delphi study
Source: Womens Health (Lond). 2025 Oct 16;21:17455057251378957. doi: 10.1177/17455057251378957 (PMC12536094; doi:10.1177/17455057251378957)
Supplement: sj-pdf-2-whe-10.1177_17455057251378957 – Supplemental material for Research priorities in vulvodynia: A modified Delphi study [file sj-pdf-2-whe-10.1177_17455057251378957.pdf]

e-Delphi Survey – Phase 1

Thank you for agreeing to take part in our research to identify research priorities in vulvodynia.

We are interested to hear about issues affecting you as a person diagnosed with vulvodynia, a healthcare professional involved in clinical care of patients with vulvodynia, a researcher with expertise in vulvodynia research, or a person who works with or on behalf of people with vulvodynia in another capacity, for example within a charity or support organisation.

Which of the following best describes you? (Select all that apply)

1. A person with a formal diagnosis of vulvodynia from a healthcare professional
2. A healthcare professional with experience in providing care to patients with vulvodynia
3. A researcher with expertise in vulvodynia
4. An individual working with or on behalf of people with vulvodynia in another capacity

[Based on participants' responses to this question, they will be directed to answer one of the following sets of follow-up questions]

1. A person with a formal diagnosis of vulvodynia from a healthcare professional

- How long ago were you diagnosed with vulvodynia?
- For how long have you experienced symptoms of vulvodynia?
- To what extent does vulvodynia impact your life?

2. A healthcare professional with experience in providing care to patients with vulvodynia

- Which of the following best describes your occupation:
  - General practitioner
  - Specialist nurse / Nurse practitioner
  - Gynaecologist
  - Urologist
  - Physiotherapist
  - Pain specialist
  - Sexual health specialist
  - Psychologist or other mental health professional
  - Other (please specify)
- For how long have you been involved in or had experience of providing care to individuals with vulvodynia? Care could include diagnosis, treatment, referral, etc.
- How frequently do you encounter cases of vulvodynia in your healthcare practice?
  - Rarely or never
  - Occasionally
  - Sometimes
  - Frequently
  - Very often or always / I am a vulvodynia specialist

3. A researcher with expertise in vulvodynia

- Which of the following best describes your academic or research background?
  - Medical and health sciences
  - Psychology and psychiatry
  - Sociology or other social science background
  - Epidemiology
  - Public health
  - Health economics
  - Other (please specify)
- Which of the following describes the kind of research related to vulvodynia that you do? You can select more than one response.
  - Preclinical research
  - Epidemiological research
  - Diagnostic studies
  - Clinical trials
  - Psychosocial research
  - Qualitative research
  - Health services research
  - Translational research
  - Community-based / participatory research
  - Other (please specify)
- For how long have you been doing research related to vulvodynia?

4. An individual working with or on behalf of people with vulvodynia in another capacity

- In what capacity do you work with or on behalf of individuals with vulvodynia?
  - Support worker
  - Charity worker
  - Policymaker
  - Other (please specify)
- For how long have you worked in this capacity?

In your opinion, what are the most important questions/problems related to vulvodynia that research could address? What are your reasons for selecting this/these question(s)/problem(s)? You can provide up to five responses.

For example, your responses might be related to causes, diagnosis, or treatment of vulvodynia, health and health-related quality of life, mental health and wellbeing, relationships for people living with vulvodynia, or another topic not listed here.

Suggestion 1 and reason

Suggestion 2 and reason

Suggestion 3 and reason

Suggestion 4 and reason

Suggestion 5 and reason

Where do you live?

What is your age in years?

